# Guide to assembling a successful K99/R00 application

**DOI:** 10.1017/cts.2023.639

**Published:** 2023-09-29

**Authors:** Peter Michaely, Suzette J. Bielinski, Kenneth Campbell, Franco D’Alessio, Delphine Dean, Yumei Feng Earley, Robert Paine, Guy Salama, Inga Peter

**Affiliations:** 1 Department of Cell Biology, UT Southwestern Medical Center, Dallas, TX, USA; 2 Division of Epidemiology, Department of Quantitative Health Sciences, Mayo Clinic College of Medicine and Science, Rochester, MN, USA; 3 Division of Cardiovascular Medicine, University of Kentucky, Lexington, KY, USA; 4 Division of Pulmonary and Critical Care Medicine, Johns Hopkins University, Baltimore, MD, USA; 5 Department of Bioengineering, Clemson University, Clemson, SC, USA; 6 Department of Pharmacology, Center for Molecular and Cellular Signaling in the Cardiovascular System, University of Nevada, Reno, NV, USA; 7 Division of Respiratory, Critical Care and Occupational Pulmonary Medicine, University of Utah, Salt Lake City, UT, USA; 8 Department of Medicine, University of Pittsburgh, Pittsburgh, PA, USA; 9 Department of Genetics and Genomic Sciences, Icahn School of Medicine at Mount Sinai, New York, NY, USA

**Keywords:** K99/R00, pathway to independence

## Abstract

The National Institutes of Health’s (NIH) K99/R00 Pathway to Independence Award offers promising postdoctoral researchers and clinician-scientists an opportunity to receive research support at both the mentored and the independent levels with the goal of facilitating a timely transition to a tenure-track faculty position. This transitional program has been generally successful, with most K99/R00 awardees successfully securing R01-equivalent funding by the end of the R00 period. However, often highly promising proposals fail because of poor grantsmanship. This overview provides guidance from the perspective of long-standing members of the National Heart, Lung, and Blood Institute’s Mentored Transition to Independence study section for the purpose of helping mentors and trainees regarding how best to assemble competitive K99/R00 applications.

The purpose of the National Institutes of Health (NIH) K99/R00 grant program is to increase the number of early-stage investigators by supporting the transition from the postdoctoral level to the tenure-track assistant professor level. The core milestone of achievement is the ability of the new investigator to secure R01-equivalent funding by the end of the R00 phase. All 21 NIH institutes support K99/R00 grants with the largest dollar allocations from the National Cancer Institute (NCI), the National Heart, Lung, and Blood Institute (NHLBI), and the National Institute of Aging (NIA) (Fig. 1). Of these three institutes, the NHLBI has been the most consistent in both success rate and grant dollars allocated (Fig. 2). Most K99/R00 awardees successfully find tenure-track positions, and the majority secure R01-equivalent funding by the end of the R00 period [1,2] (Fig. 3); however, the authors, long-term standing reviewers for the NHLBI Mentored Transition to Independence (MTI) study section, observed many promising applications that failed because of poor grantsmanship. Poor grantsmanship is in part an insufficient response to the distinctive requirements of this funding mechanism coupled with a failure of mentoring. The ever-increasing demands on faculty [3] coupled with the need to maximize research productivity may be curtailing the ability of some faculty to provide the same degree of mentoring as was given in past years. Also, clear directions and expectations for the mentor’s role in the application process may be lacking. Therefore, the purpose of this article is to provide guidance from the perspective of long-standing members of the NHLBI study section that reviews K99/R00 applications both to facilitate the ability of mentors to critique trainee applications and to maximize the ability of trainees to assemble competitive applications given the unique nature of the K99/R00 grant mechanism.

**Figure 1.**
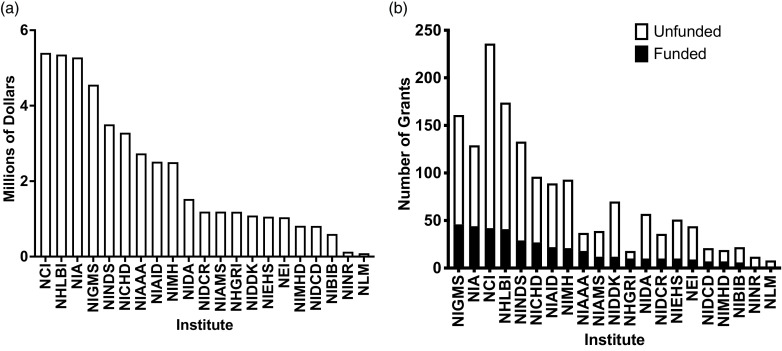
(***a***) Dollars allocated to the K99 vehicle in FY2022 [7]. (B) K99 grants funded in FY2022 [7]. NIH institute abbreviations are as follows: NCI = National Cancer Institute; NHLBI = National Heart, Lung, and Blood Institute; NIA = National Institute of Aging; NIGMS = National Institute of General Medical Sciences; NINDS = National Institute of Neurological Disorders and Stroke; NICHD = National Institute of Child Health and Human Development; NIAAA = National Institute of Alcohol Abuse and Alcoholism; NIAID = National Institute of Allergy and Infectious Diseases; NIMH = National Institute of Mental Health; NIDA = National Institute on Drug Abuse; NIDCR = National Institute of Dental and Craniofacial Research; NIAMS = National Institute of Arthritis and Musculoskeletal and Skin Diseases; NHGRI = National Human Genome Research Institute; NIDDK = National Institute of Diabetes and Digestive and Kidney Diseases; NIEHS = National Institute of Environmental Health Sciences; NEI = National Eye Institute; NIMHD = National Institute on Minority Health and Health Disparities; NIDCD = National Institute on Deafness and Other Communication Disorders; NIBIB = National Institute of Biomedical Imaging and Bioengineering; NINR = National Institute of Nursing Research; NLM = National Library of Medicine.

**Figure 2.**
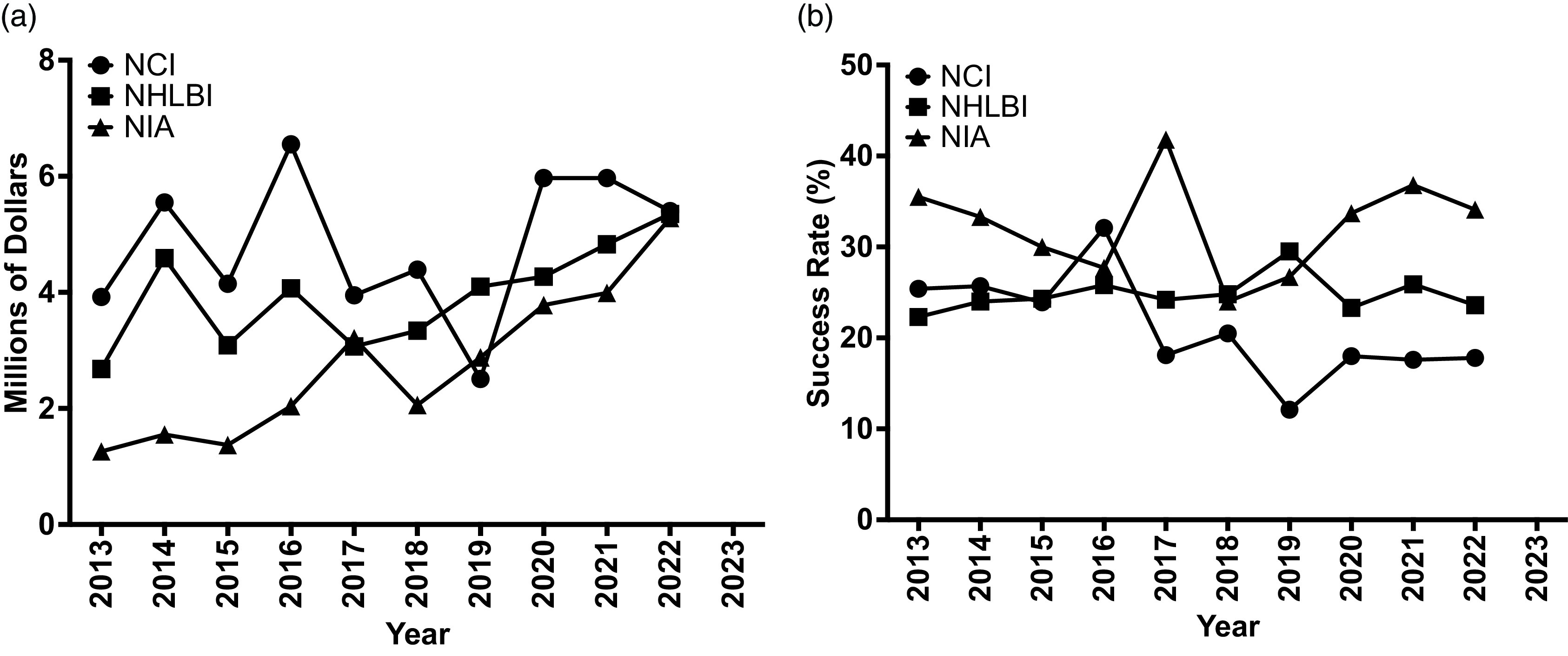
(***a***) History of grant dollars allocated to the K99/R00 grant vehicle for the three NIH institutes (NCI, NHLBI, and NIA) with the greatest number of grant dollars allocated in FY2022 [7]. (***b***) Success rates, as defined as percent of applications funded, is shown for K99/R00 grant applications to the NCI, NHLBI, and NIA from FY2013-2022 [7]. NCI = National Cancer Institute; NHLBI = National Heart, Lung, and Blood Institute; NIA = National Institute of Aging; NIH = National Institutes of Health.

**Figure 3.**
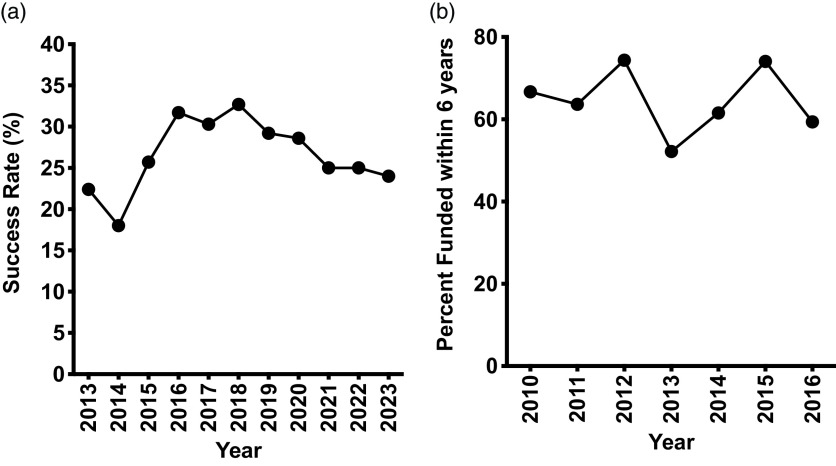
(***a***) Success rate of R01 submissions at the NHLBI by calendar year as assessed and recorded in NIH RePORTER (https://reporter.nih.gov). (***b***) The success of NHLBI K99/R00 awardees in obtaining R01-equivalent funding within 6 years of the K99 notice of award. Dates reference the year in which the K99 notice of award was issued as recorded in NIH RePORTER (https://reporter.nih.gov). R01-equivalent grant awards include all awards that provide at least $200,000 annually in direct support for multiple years. NHLBI = National Heart, Lung, and Blood Institute; NIH = National Institutes of Health.

## Overview of the K99/R00 Evaluation Process

K99/R00 applications are reviewed based upon five components: candidate, career development plan (CDP), research plan, mentoring, and institution/environment. Each component is essential, and a moderate weakness in any one component can doom an application. Of the five components, many reviewers consider the candidate component as most important, in part because the purpose of the K99/R00 is to develop independent investigators. The research plan is a close second and needs to demonstrate that the candidate can ask important questions and that the proposed area of research will provide a niche within which the candidate can build a productive career. Next in importance is the CDP, which needs to identify specific needs for additional training and training areas and detail how the candidate will acquire the training needed to become an independent investigator. The mentor and institution/environment components are both essential, but reviewers commonly evaluate these components on a competency level. Will the mentor and mentoring team provide the training and commitment necessary to facilitate the transition of the applicant into an independent, tenure-track position? For institution/environment, the essential question is will the research environment and the institution adequately support the applicant’s needs during the training phase?

## Candidate

The candidate component needs to discuss the applicant’s career objectives, their scientific accomplishments, and how they will separate scientifically from their mentor. The career objectives need to be more than "I want to be an independent investigator." That goal is assumed for all applicants. Instead, the objectives need to explain why the applicant is pursuing an academic career and how the applicant plans to make their mark in research. The scientific accomplishments should be broken into sections, based on either scientific themes or academic phases. Each section should include a description of published work, funding, and professional honors, awards, and accomplishments. Funding that matters most for reviewers are grants awarded directly to the candidate whether public (F32, F99, etc.) or private (foundations, industry, etc.). Reasons should be provided if the training period was longer than is typical or if the applicant did not change mentors following graduate training.

How the applicant will separate scientifically from the mentor is an essential element. The applicant needs to detail how this separation will occur and how the research in the R00 phase will provide a productive independent niche. The mentor’s letter must corroborate and affirm the separation plan. If the mentor’s letter is deficient in this regard, the applicant’s statements can be dismissed as wishful thinking, particularly if the mentor has a history of grant funding on the topic. It should also be clear in both the CDP and the research plan that the applicant will use the skills obtained in the K99 phase to address different questions than those pursued by the mentor during the R00 phase. The K99/R00 is a transition-to-independence award, and the ability of the candidate to operate independently is of paramount importance.

The second critical element is, as it has long been [4], the number and quality of first-authored, peer-reviewed, research papers. Papers published in high-profile journals are not required, but candidates with strong applications normally have at least one first-authored, mid-tier-or-better, peer-reviewed publication from both their graduate and postdoctoral training phases. Middle-authored publications, reviews, and publications in low-impact journals are given much lower consideration. What constitutes high-profile, mid-tier, and low-impact varies by field and aggregate impact factor is not a primary criterion. Most important is sustained productivity in journals that are read and respected in the field associated with the research proposal. Applicants should provide an explanation for any gaps in their publication record.

Reviewers emphasize first-authored (and co-first-authored) research papers for two principal reasons. First, reviewers have more confidence that a first author was involved in most of the work and has the skills to do similar work in the future. Past productivity is among the strongest predictors of future productivity [5]. Second, reviewers ask, with the training present in the CDP and a solid publication from the K99 period, would the applicant be competitive for a tenure-track position, which is required for transitioning to the R00 phase? If the answer is no, the score for the candidate component will be poor.

More rarely, applicants with outstanding publication records can also be questioned. If the existing publication record is so strong that the applicant is already competitive for a tenure-track position, reviewers will rightly question the need for the K99 phase, and hence whether a K99/R00 is appropriate for the candidate. To counter this concern, the CDP of such applications needs to clearly justify why additional training is needed.

Reviewers value publications from the postdoctoral period (and in particular, publications co-authored with the current mentors) more highly than those in the graduate period. Postdoctoral publications are usually more aligned with the research goals of the K99/R00 and thus better demonstrate the ability of the applicant to perform the proposed work. Moreover, postdoctoral trainees are commonly given more freedom to design research projects than graduate-level students, and thus postdoctoral productivity is viewed as a better indication of the applicant’s ability to ask questions worthy of investigation. Finally, many reviewers see an applicant who has demonstrated productivity at different career stages (graduate and postdoctoral) as likely to also be productive in the R00 independent phase. An applicant with a strong publication record in the postdoctoral phase is viewed as being on an upward trajectory. By contrast, many reviewers consider the lack of a first-authored research publication with the postdoctoral mentor to be a moderate weakness.

The applicant’s biosketch should summarize the key points of the candidate component with a succinct description of the applicant’s past accomplishments and their path to independence. References for past publications must be provided. Moreover, applicants should clearly identify publications that are not peer-reviewed research papers, such as reviews, abstracts, oral presentation, or preprints. Parsing out peer-reviewed from non-peer-reviewed work adds unnecessary burden for the study section reviewers. A link to the applicant’s complete publication record in MyNCBI should also be provided. Elements that improve the candidate component score include graduate and postdoctoral fellowship awards, honors, examples of leadership, and any professional experience such as teaching or serving as a reviewer.

## Career Development Plan

The CDP component needs to detail both research training and career development activities. Proposed research training needs to be for skills that the applicant does not currently possess. If the applicant proposes to gain expertise in a new field, formal coursework should be detailed. Moreover, training aimed at a better understanding of pathophysiology of studied conditions is viewed favorably. Descriptions of the research training should include who will provide the training, how long the training activity will last, and how the new skills will be used in the research plan. Details of training must match statements in the letters of the mentoring component. In particular, the mentor’s letter needs to echo the same training plan as outlined in the candidate’s CDP. Any aspect of the training that is not solidly within the expertise of the mentor should have a collaborator or consultant. If a large aspect of the training is outside the expertise of the mentor, a co-mentor with that expertise should be present. Applicants should state why each member of the mentoring committee was chosen and describe how they will interact with each member. A CDP with a poor description of training necessary for the research plan can negatively impact a reviewer’s assessment of the viability of the research plan.

Many applications lack a good description of career development, and reviewers commonly consider such a lack to be a moderate to major weakness. In addition to acquiring additional skills and gaining expertise in a new field, the CDP should have career development training including topics such as teaching, mentorship, lab management, manuscript/grant writing, job interviewing/negotiation, and leadership training. An ability to teach and train students is an important aspect of running a new independent research group, and reviewers respond positively to applicants who express an interest in teaching and who have a track record of student training and mentoring. Mentors of applicants can and should provide informal training on these topics, but most reviewers view the addition of formal training in career development more favorably than informal training alone. Most institutions offer workshops on career development, and there are also national workshops such as the NIH/NMA Academic Career Development Workshop.

The CDP should conclude with a timetable that relates training to the research goals. The timetable should include well-defined milestones with regular evaluations by the mentoring committee. While training activities should end with the K99 phase, the discussion of research activities of the R00 phase is helpful for reviewers to see how the K99 training activities will be employed in the independent phase. The key is to illustrate that the CDP is focused on providing the applicant with the tools during the K99 phase that will be needed in the R00 independent phase.

## Research Plan

Like research (R) grant applications, the K99/R00 research plan has significance, innovation, and approach sections. The significance needs to (i) detail why the proposed research is important to the mission of the institute to which the grant is sent; (ii) provide a focused, but rigorous review of why the proposed work is important – that is, identify the knowledge gaps and potential impact; and (iii) explain why the applicant is well positioned to conduct the research. The innovation section needs to detail how the research plan will move the field forward through aspects such as novel hypotheses and novel methods. Plans that are seen as iterative receive poor scores. Each study in the approach section needs to have a rationale and sufficient detail for a reviewer to understand what will be done, expected results, and pitfalls/alternative approaches. Scientific rigor, including experimental detail, statistical handling of data and power calculations, must be addressed in the research plan. Strong applications have preliminary data for both the K99 and R00 phases. Alternative approaches are critical to illustrate how the applicant will pivot if the hypothesis for a study proves false.

A unique feature of the K99/R00 is the dual nature of the research plan, which covers both the K99 and R00 phases. The purposes of the K99 phase are to provide the applicant with the opportunity to exercise the new skills that will be employed in the R00 phase and to facilitate the applicant’s efforts to publish a final paper prior to searching for a tenure-track position. The purpose of the R00 phase is to establish an independent research program that can progress to an awarded research grant (R01 or equivalent) by the end of the R00 period. The quality of the R00 phase project is thus of paramount importance, yet it is this aspect that is often poorly constructed. An R00 plan that is vaguely described, dependent on research outcomes of the K99 phase, unlikely to be productive, or dependent on the mentor and/or resources at the mentor’s institution will result in poor scores.

The research plan must be tightly focused. The page limit is short. Moreover, jargon must be avoided because the members of a K99/R00 panel have diverse expertise. Figures and tables must be both essential and readable. Too commonly, research plans contain extraneous visuals or have key visuals that are too compressed to be effective. If reviewers cannot understand what is planned, the component score will be worse.

## Mentoring

Many studies have illustrated the benefits of mentoring [6], and mentoring is an essential element of the K99/R00 program [1]. Mentors must show commitment to the applicant both in the training provided during the K99 phase and by fostering the ability of the applicant to operate independently in a productive niche during the R00 phase.

The mentoring component consists of letters from the primary mentor, co-mentors, and any other individuals contributing to the training of the applicant. The mentor’s letter is of paramount importance and must detail the mentor’s evaluation of the applicant, their research qualifications, their experience in mentoring trainees to independent positions, their training plan for the applicant, their funding to support the applicant in the K99 phase, and a clear statement regarding how the applicant will separate scientifically during the R00 phase. If the applicant will be working on a topic related to the mentor’s own work, the letter should detail what aspects the applicant will take with them into the independence phase and a commitment from the mentor not to compete with the applicant on that project. Co-mentors should provide similar information, but they should emphasize those aspects most central to their responsibilities within the mentoring team. The training plan must describe both research and career development aspects. If the primary mentor is junior or otherwise lacking a strong track record of mentoring, a more experienced co-mentor should be part of the mentoring team. The division of labor between the primary mentor and co-mentors should be clearly stated. The mentor letter must also describe the plan for regular evaluation of the applicant during the K99 phase, including the metrics that will be employed. Combined mentor/co-mentor letters are acceptable, provided all components are addressed and the division of labor is clear. Letters from collaborators, contributors, and consultants should be provided, and these letters need to detail their expertise and contributions to the training of the applicant. Biosketches must be included for each member of the mentoring team. Applicants should review all letters and biosketches for accuracy and consistency. Of key importance is that the training described in the mentoring component is echoed in the CDP. Many reviewers view the lack of concordance between the CDP and the mentoring component as a moderate weakness.

Other aspects of the application can impinge upon the score for the mentoring component. Reviewers expect mentors to provide guidance to applicants as they assemble their applications. Strong grantsmanship is an indication that mentors provided the applicant with constructive feedback. Poor grantsmanship is an indication that mentors are disengaged and are thus less likely to expend the time and effort needed to optimally launch the applicant’s transition to independence.

## Institution/Environment

The institution/environment component needs to document the institutional support for the K99 training needs, including both research and career development aspects. The letter from the department or division chair should be personalized for the applicant, provide an overview of the available resources, and clearly state that the applicant will be able to devote at least 75% of the time to research activities with the remaining time devoted to career development. If the applicant is a noncitizen, the letter must support the applicant’s visa requirements during the K99 phase. A commitment to the applicant in the form of promotion or consideration for a tenure-track position is not required but can improve the component score, particularly if this commitment is not tied to whether the applicant is awarded a K99/R00.

## Final Impact Score

Reviewers evaluate each component separately and then compile a final impact score. Yet, there is a considerable interplay between various scored components of the K99/R00 application. For example, a poor CDP can negatively reflect the mentor/mentoring team as well as the candidate’s potential for success. Thus, a poor CDP can contribute to three different scored components. Also, the final impact score is not the average of the component scores. Rather the final impact score is a reflection of the number and character of the weaknesses identified in each component. Reviewers give a final impact score of 1, 2, or 3 for applications that have only a few minor weaknesses. If any component has a moderate weakness, the final impact score will normally not be stronger than a 4. If any component has a major weakness, the final impact score will normally be in the 7–9 range, even if all other aspects are superlative.

## Additional Review Criteria

In addition to the five components described above, K99/R00 applications also require candidates to detail training in the responsible conduct of research, and as applicable, details regarding the protection of human subjects, protection of vertebrate animals, biohazards, select agents, resource sharing, and authentication. If human or animal subjects are part of the proposal, sex as a variable must be addressed. While these additional components are not given a numerical score, deficiencies in these criteria can dampen reviewer enthusiasm. Particular attention should be given to human subjects, vertebrate animals, and biohazards because these components are discussed prior to the submission of final impact scores. Applicants should not try to circumvent page limitations by including important experimental design features in the animal component. For example, scientifically important issues such as sex as a biological variable should be included in the research plan. Deficiencies in these components can cause reviewers to question the ability of an applicant to perform the work proposed in the research plan.

## Who Should Apply and to Which Institute

The K99/R00 is intended to facilitate the transition of postdoctoral fellows from a mentored environment into an independent, tenure-track position. Most competitive applications are submitted by candidates who are in their third or fourth year of postdoctoral training and who have at least one first-authored, peer-reviewed research paper accepted for publication with their current mentor. Strong applications also have clear training needs that the K99 will address and a well-described research program for the R00 phase. While most K99/R00 applicants have a PhD degree, any candidate with a graduate-level clinical or research doctorate degree (e.g., MD, DDS, DVM, etc.) is eligible and candidates with uncommon academic credentials are given careful consideration.

Each institute supports the K99/R00, and candidates should select the institute whose goals most closely align with the specific aims of the application. Institute goals overlap, and when this occurs, candidates may wish to consider other factors such as pay line and average dollars provided per funded grant (Fig. 1). Consistency of past support for the K99/R00 (Fig. 2) may provide an indication of future support for the vehicle.

The K99/R00 vehicle is not the only mechanism that can support a transition to independence, but also other mechanisms should be considered based on eligibility. Of the many K awards available, the panels that review K99/R00 applications also review K22 and K25 applications. The K22 program is intended for “intramural” candidates, working at the NIH, who wish to transition to extramural, tenure-track positions. The K22 award is essentially the intramural counterpart to the K99 and, like the K99, the K22 has an R00 phase subsequent to the training phase. The K25 program is intended for candidates with quantitative backgrounds (e.g., computer science or engineering) who are seeking to transition into biological or medical science. The K25 differs from the K99 in that the K25 is a 5-year training award without an R00 phase, has a higher pay line, allows for a larger budget, and has different eligibility requirements. K25 applicants can not only be postdocs, but they can also already hold a full-time tenure/tenure-track faculty appointment. Of note, unlike K99 applicants, K25 applicants must be a citizen or a noncitizen national of the USA or have been lawfully admitted for permanent residence by the time of the award.

On a final note, it is important to reiterate that the K99/R00 is a training award. If a candidate is ready to apply for a job, they should forego the K99, get their job, and apply for an R01. Early-stage investigators receive a bonus to their percentile score for new R01 submissions at many NIH institutes, making the success rate about the same as that for K99/R00 awards (Figs. 2 and 3). An R01 provides more funds, has a longer duration, and is viewed with greater favor by institutional promotion and tenure committees than an R00. Career advancement is frequently tied to success in obtaining R01-equivalent grants, and the sooner an assistant professor can demonstrate sustained success, the better chance they have of obtaining tenure.
